# Forces from Stochastic Density Functional Theory under Nonorthogonal Atom-Centered Basis Sets

**DOI:** 10.1021/acs.jctc.1c00794

**Published:** 2022-01-31

**Authors:** Ben Shpiro, Marcel David Fabian, Eran Rabani, Roi Baer

**Affiliations:** † Fritz Haber Center for Molecular Dynamics and Institute of Chemistry, The Hebrew University of Jerusalem, Jerusalem 9190401, Israel; ‡ Department of Chemistry, University of California, Berkeley, California 94720, United States; ¶ Materials Sciences Division, Lawrence Berkeley National Laboratory, Berkeley, California 94720, United States; § The Raymond and Beverly Sackler Center of Computational Molecular and Materials Science, Tel Aviv University, Tel Aviv 69978, Israel

## Abstract

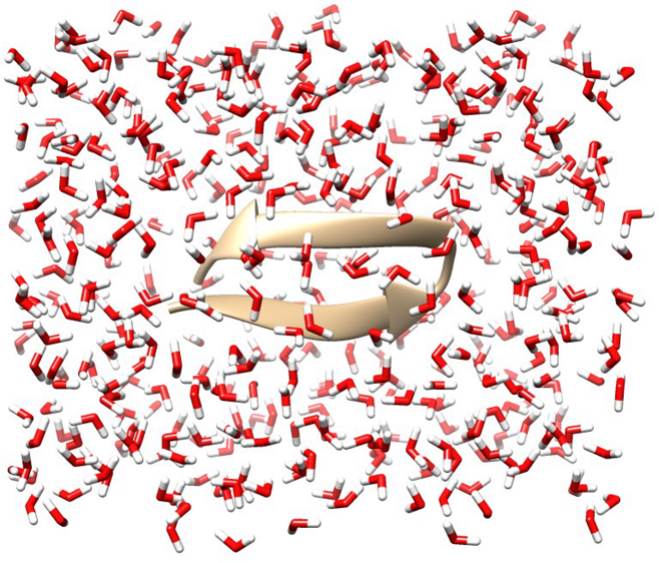

We develop a formalism for calculating forces on the nuclei within the linear-scaling stochastic density functional theory (sDFT) in a nonorthogonal atom-centered basis set representation (Fabian et al. Wiley Interdiscip. Rev.: Comput. Mol. Sci.2019, 9, e1412, 10.1002/wcms.1412) and apply it to the Tryptophan Zipper 2 (Trp-zip2) peptide solvated in water. We use an embedded-fragment approach to reduce the statistical errors (fluctuation and systematic bias), where the entire peptide is the main fragment and the remaining 425 water molecules are grouped into small fragments. We analyze the magnitude of the statistical errors in the forces and find that the systematic bias is of the order of 0.065 eV/Å (∼1.2 × 10^–3^*E*_*h*_/*a*_0_) when 120 stochastic orbitals are used, independently of system size. This magnitude of bias is sufficiently small to ensure that the bond lengths estimated by stochastic DFT (within a Langevin molecular dynamics simulation) will deviate by less than 1% from those predicted by a deterministic calculation.

## 1 Introduction

Kohn–Sham density functional theory (KS-DFT) is often used for estimating the forces on the nuclei in *ab initio* molecular dynamics simulations, with which reliable predictions concerning structure and properties of molecules can be made. Despite the fact that it can be used to study extended molecular systems relevant to biomolecular chemistry and materials science,^1−4^ the conventional applications are limited in size due to the cubic algorithm complexity. Therefore, several approaches to KS-DFT have been developed and are routinely used for treating such extended systems. These include linear-scaling approaches which rely on electron localization within the system’s interior volume,^5−33^ or the tight-binding DFT approach, which uses a very small basis set complemented by approximations calibrated with empirical data,^34−36^ and the orbital-free DFT, which is applicable to relatively homogeneous systems.^37,38^ The way many of the linear scaling approaches achieve their gentle algorithmic complexity involves imposing a sparse structure on the KS density matrix (DM) in a local real-space basis representation, effectively truncating the protruding elements. The rationale of this procedure relies on the electron localization which characterizes many large systems.^39^ However, in metallic systems at low-temperature and for low band semiconductors, the localization length is very large, and such approaches are difficult to apply.^17^

In order to enable treatment of systems in which electron coherence is nonlocal, a different linear scaling approach was proposed and dubbed stochastic density functional theory (sDFT).^40^ In sDFT, we use a sparse representation of the KS-DM which does not rely on truncation or modification of its elements. Instead, sDFT is based on the paradigm that the expectation values of the system observables can be regarded as random variables in a stochastic process with an expected value and a fluctuation. The fact that estimation of electronic structure quantities can be done by statistical sampling allows for a natural and highly effective implementation of sDFT on parallel architectures.

The source of errors in sDFT is statistical in nature and involves fluctuations, the magnitude of which can be controlled by statistical sampling theory and/or by variance-reducing techniques, such as the embedded-fragment method,^41−44^ or the energy windowing approach.^45,46^ In addition to statistical fluctuations, the sDFT estimates of the electron density and the forces exhibit bias errors resulting from the nonlinear nature of the SCF iterations.^44,47^ The magnitude of the bias can be controlled by using the above-mentioned variance-reducing techniques.

Early implementations of sDFT were based on real-space grid representations of the electron density^40,42,43,47,48^ and applied to relatively homogeneous systems: either to pure bulk silicon,^43,47^ silicon with impurities,^46^ H–He mixtures^49^ or to finite-sized hydrogen-passivated silicon nanocrystals and water clusters.^42,50,51^ We recently demonstrated that the noisy forces produced by sDFT in the real-space grid representation can be used within a Langevin dynamics approach, to determine structural properties of such large systems.^42,52^

The real-space implementation of sDFT is especially useful as a starting point for postprocessing DFT-based methods, such as the stochastic GW for charge excitations,^53,54^ the stochastic time-dependent DFT and Bethe-Salpeter equations for neutral excitations,^55−57^ and for conductance calculations in warm dense matter.^49^

If one is only interested in the ground state atomistic structure, real-space grid representation could be quite expensive, and a more efficient representation may be beneficial. For this purpose, we recently developed an sDFT approach based on nonorthogonal atom-centered basis sets.^44^ We found that the Hamiltonian within this compact basis has a much smaller energy range than in the real-space grid, allowing a significant speedup of sDFT calculations.

Despite the fact that sDFT with the nonorthogonal atom-centered basis set is designed to address the structural properties of large systems, up to now, we did not have the capability to estimate the forces on nuclei and therefore focused only on the electronic energy and density of states.^44^ In this paper, we develop the necessary theory and computational tools for calculating the forces while maintaining the linear-scaling complexity of sDFT. In addition, we analyze the statistical fluctuations and the biases in the forces, using as a benchmark the heterogeneous system of the Tryptophan Zipper 2 (Trp-zip2) peptide solvated in water.

The manuscript is organized as follows: In Section 2, we introduce our formalism for the stochastic forces calculations. Then, in Section 3, we present the benchmark calculations on the Tryptophan Zipper 2 (Trp-zip2) peptide in solution. Finally, we summarize and discuss the results in Section 4.

## 2 Force Calculations in Stochastic Density Functional Theory

In this section, we describe the theory of the electronic forces on nuclei within the finite temperature KS-DFT formalism. We set the notations and describe the basis set representation we use for Kohn–Sham DFT in subsection 2.1 with the combined implementation using real space grids briefly described in subsection 2.2. Expressions for the forces are given in subsection 2.3 with a detailed derivation given in Appendix A. Finally, in subsections 2.4 and 2.5, we provide the details behind the stochastic evaluation of the electronic density and any other observables in sDFT (including the forces) and present the statistical errors involved.

### 2.1 Setting the Stage

The KS Hamiltonian is given by
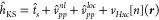
1where *t̂*_*s*_ = −(1/2)∇^2^ (we use atomic units throughout the paper) is the electron kinetic energy operator, and *v̂*_*pp*_^*nl*^ = ∑_*C*∈*nuclei*_*v̂*_*pp*(*C*)_^*nl*^, and *v̂*_*pp*_^*loc*^ = ∑_*C*∈*nuclei*_*v*_*pp*(*C*)_^*loc*^(***r*^** – ***R***_*C*_) are the nonlocal and local norm-conserving pseudopotential terms in the Kleinman-Bylander form,^58,59^ for nucleus *C*, at position ***R***_*C*_. The last potential term, *v̂*_*Hxc*_, is the Hartree and exchange correlation potential, depending on the electron density, *n*(***r***)
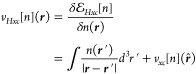
2where  is the Hartree and exchange-correlation energy functional.

We use a nonorthogonal atom-centered basis set, ϕ_α_(***r***), α = 1,...,*K*, with an overlap matrix *S*_*αγ*_ = ⟨ϕ_α_|ϕ_γ_⟩, α, γ = 1,...,*K*. Within such a basis set approach, the *K* × *K* DM is given as an operator involving a function of *HS*^–1^
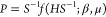
3where *H*_*αγ*_ = ⟨ϕ_α_|*ĥ*_KS_|ϕ_γ_⟩ and
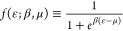
4is the Fermi–Dirac distribution function. The DM is used to calculate expected values of single-electron observables *ô* as
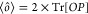
5where *O* is the matrix representing *ô* in the basis, with elements
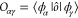
6and the factor of 2 accounts for the electron’s spin in a closed shell representation. For example, the expectation value of the density operator *n̂*(***r***) is the electron density, given by
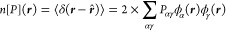
7The DM in eq 3 minimizes the total electronic free-energy:
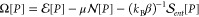
8Here,  is the electronic internal energy
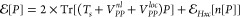
and the number of electrons is given by
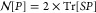
The actual value we use for the chemical potential μ is tuned to enforce  to be equal to the actual number of electrons in the system (see ref (44) for detail). Finally,  is the entropy of the noninteracting electrons of the KS system, given by
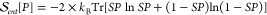
Equations 1–7 must be solved together, and the resulting solution for the density *n*(***r***) and the DM *P* is called the self-consistent field (SCF) solution to the KS equations. The procedure for reaching SCF solution is iterative: in each iteration, called an SCF cycle, *P* is calculated from *H* using eq 3, *n*(***r***) is from *P* from which *v*_*Hxc*_[*n*](***r***) is calculated, and a new KS Hamiltonian matrix *H* is built.

### 2.2 Combined Real-Space Grid and Basis Set Implementation

The theory described in the section above uses, in addition to the basis function ϕ_α_(***r***), also a Cartesian grid (with uniform grid-spacing *h*) which spans the space occupied by the electron density. The grid is used to evaluate the matrix elements of eq 6 of various observables *ô*, expressible as operators on the grid
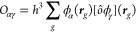
9where ***r***_*g*_ are the grid points (*g* is a 3D index). Each matrix element of eq 9 can be evaluated efficiently, while we can also gain by parallel architecture, allowing different cores to independently compute different *αγ* pairs. [This requires a fast evaluation of basis functions ϕ_α_(***r***_*g*_) at the grid points. For this, we employ standard quantum-chemical Cartesian functions, expressible as sums of triple products, ϕ_α_(*x*, *y*, *z*) = ∑_*p*_ξ_α_^*p*^(*x*)η_α_^*p*^(*y*)ζ_α_^*p*^(*z*) where the sums of ξ_α_^*p*^(*x*), η_α_^*p*^(*y*), and ζ_α_^*p*^(*z*) are the primitive functions of the basis. At grid point ***r***_*g*_, the basis function is a sum (over the primitive functions) of triple products formed from three 1D vectors: ξ_α_^*p*^(*x*_*g*_), η_α_^*p*^(*y*_*g*_), and ζ_α_^*p*^(*z*_*g*_) which are kept in memory. The same technique is used for the evaluation of the derivatives of the basis functions, which is relevant for the calculation of forces, see Supporting Information, Section S1.] In particular, the pseudopotentials *v̂*_*PP*_^*nl*/*loc*^ are such grid operators. Evaluating the electron density of eq 7 at the grid points allows calculation of the density-dependent Hartree and XC potentials. For the former, we use fast Fourier transform techniques.^60^

### 2.3 Electronic Forces on the Nuclei

In this subsection, we give formal expressions for the electronic forces on the nuclei expressible as matrix trace operations, based on a finite temperature formalism presented in Appendix A. Our derivation and final results are similar yet differ in many ways with those of ref (61). We calculate the work done by the electrons as nucleus *C* is displaced by δ_*C*_*X* in the *x*-coordinate. This work is the change in the free energy of eq 8, and therefore
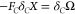
10where *F*_*C*_ is the *x*-component of the force on the displaced nucleus. The atom displacement δ_*C*_*X* has three types of effects: it causes an explicit change in its contribution to the pseudopotential *v̂*_*pp*_^*nl*/*loc*^ → *v̂*_*pp*_^*nl*/*loc*^ + δ_*C*_*v̂*_*pp*_^*nl*/*loc*^, it displaces the basis functions ϕ_α_ → ϕ_α_ + δ_*C*_ϕ_α_, and it induces a variation in the DM, *P* → *P* + δ_*C*_*P*, since *P* is required to be the minimizer of the free energy. Note that due to this minimum principle δ_*C*_Ω is unaffected (to first order) by δ_*C*_*P* so that the work done on the atom (see Appendix A)
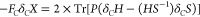
11is given solely in terms of the variations in the Hamiltonian
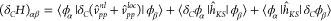
12and the overlap
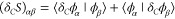
13matrices. The first term in eq 12 is the explicit change in the pseudopotential, giving the direct forces on the atom. The second and third terms in δ_*C*_*H* (and similar terms in eq 13 for δ_*C*_*S*) are due to the variation in basis functions, and they lead to the so-called Pulay forces,^62^ on the atom. More details concerning the calculation of (δ_*C*_*S*)_*αβ*_ and (δ_*C*_*H*)_*αβ*_ are given in Supporting Information, Section S1.

The estimation of the expectation value of a one-body observable *ô*, given by eq 5, requires the calculation of the trace of the matrix *OP*. By definition Tr[*OP*] = ∑_*k*=1_^*K*^(*u*^*k*^)^*T*^*OPu*^*k*^ where *u*^*k*^ is a set of *K* orthogonal unit vectors, and the numerical effort involves *K* applications of *OP* on a vector *u*, each of which scales quadratically, and thus the overall effort scales as *O*(*K*^3^).

One essential component in reducing the scaling of this step is to exploit the sparsity of the *S*^–1^*H* operation on a vector *v*, which is used within a Chebyshev expansion,^63^ as a Fermi–Dirac function representing *P* (see eq 3). [The application of *S*^–1^ on a column vector involves repeated applications of *S* on the vector, within the preconditioned conjugate gradient method, implemented in the HSL-MA61 code. HSL is a collection of FORTRAN codes for large scale scientific computation (http://www.hsl.rl.ac.uk/).] This leads to the following method for applying the DM onto a vector *v*(8,64)
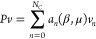
14where *v*_*n*_, *n* = 0,1,... is obtained recursively
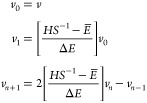
15Here,  is a shifted-scaled operator with eigenvalues in the interval [−1,1] (So *ΔE* is equal to half the spectral range, and *E̅* is its center.). The expansion coefficients depend on β and μ characterizing the Fermi–Dirac function; they rapidly decay to zero once *N*_*C*_ exceeds a system size independent value determined by *βΔE*. With this technique, the step *Pu*^*k*^ involves a linear scaling effort, and since there are *K* such vectors, the complexity of the trace operation Tr[*OP*] is reduced from *O*(*K*^3^) to *O*(*K*^2^).^65^

### 2.4 Stochastic Estimation of Observables and Forces

In order to further reduce the numerical effort to linear scaling, we use a stochastic vector approach, where the trace is sampled using *I* stochastic vectors instead of *calculated* using a complete set of *K* orthonormal vectors. The calculation effort is reduced from *O*(*K*^2^) to *O*(*IK*), and *I* is system independent. A full exposition of the method is given in ref (44). Here, we briefly mention the essential elements.

Stochastic vectors, χ^*T*^ = (χ^1^,...,χ^*K*^), have *K* random components, χ^*k*^; each is a random variable taking the values ±1 with equal probability. We refer the reader to Section S2 of the Supporting Information for definition and discussion of random variables (collectively denoted *r*), their expected values E[*r*], their variance Var[*r*], and the statistical methods for evaluating these quantities using finite samples. For each component of the stochastic vector, (1) |χ^*k*^| = 1 (2) E[χ^*k*^] = 0, and therefore, Var[χ^*k*^] = 1. Furthermore, the product χ^*k*^χ^*j*^ of any pair of components has a zero expected value (E[χ^*k*^χ^*j*^] = 0, *k* ≠ *j*), and hence, in matrix form
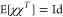
16where Id is the *K* × *K* identity matrix. We view eq 16 as the “stochastic resolution of the identity”, and using it, we express the trace of the matrix *OP* as Tr[*OP*] = Tr[*OP*E[*χχ*^*T*^]] = E[Tr[*OPχχ*^*T*^]], which upon rearrangement gives the stochastic trace formula:^66^
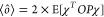
17The expected value E[χ^*T*^*OPχ*] can be estimated using a sample of size *I* with
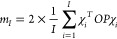
18which establishes a 70% confidence interval [*m*_*I*_ – σ_*I*_, *m*_*I*_ + σ_*I*_] for ⟨*ô*⟩ where
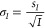
19and  is the standard deviation. We would like to highlight that since eq 18 is an average over *i* independent χ_*i*_^*T*^*OPχ*_*i*_ terms, the computation is easily implemented to gain from parallel architecture.

We can use the stochastic trace to estimate the electron density at each grid point, based on eq 7. For this, we define stochastic orbitals which are stochastic linear combinations of the basis functions, defined on the grid as
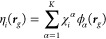
and *projected* stochastic orbitals
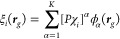
Using the above, we can now calculate the center of the confidence interval for the electron density at point ***r***_*g*_ as the sample mean:
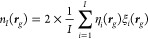
20In ref (44) we have presented CPU times showing linear scaling in the calculation of sDFT observables.

The above technique can be used to evaluate the electronic forces on the nuclei as they too are formulated as matrix traces (see eq 11). The computational effort for evaluating the direct forces coming from *v̂*_*pp*_^*nl*^ (the non-local pseudopotential) as well as all Pulay terms, for each degree of freedom, are independent of the system size since they are local (See Supporting Information Section S1.C. for detail). The computational effort for evaluating the force coming from *v̂*_*pp*_^*loc*^ (the local pseudopotential), for each degree of freedom, will scale linearly unless specialized particle mesh methods (beyond the scope of this paper) are used.

The SCF cycle of KS theory in sDFT involves using our best estimate for the density, i.e., *n*_*I*_(***r***), to build the Hamiltonian. Since *n*_*I*_(***r***) includes an uncertainty (a fluctuation), the resulting Hamiltonian matrix *H* also has a fluctuation. Then, plugging *H* into the Chebyshev expansion from which a new *n*_*I*_(***r***) is calculated converts the fluctuation into a bias, as discussed in Section S2.C of the Supporting Information. Thus, after the SCF converges, all expectation values have both an uncertainty σ_*I*_ and a bias error, which we define as
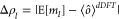
The estimation of the uncertainty σ_*I*_ can be done using eq 19, but the estimation of *Δρ*_*I*_ is more complicated since we need to determine E[*m*_*I*_]. We discuss this issue when we determine the bias error in the force (see Section 3).

### 2.5 Embedded Fragments Approach

In order to mitigate the fluctuation and bias errors, we developed a basis set version of the embedded-fragment (EF) approach,^41−44^ which can be described in a general way as introducing a correction term to the sDFT calculation. We first split all the atoms in the system into *F* fragments, such that each atom, and all basis functions centered on it, belong to one and only one fragment. If the fragments are chosen such that their size is independent of the total system size, with sublinear scaling and minimal increase in computation time we can calculate the electron density in each fragment, using 1.) deterministic DFT *n*_dDFT_^*f*^(***r***) (*f* = 1,...,*F*) and 2.) stochastic DFT *n*_*I*_^*f*^(***r***). We then use the difference
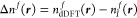
21as a correction to the sDFT calculation of the density *n*_*I*_(***r***) on the entire system:
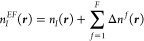
22We note that the correct result, *n*_*I*_^*EF*^(***r***) = *n*_*dDFT*_(***r***), is obtained in two limits: 1) when *F* = 1 (i.e., the entire system is a fragment) and 2) when *I* → ∞, so *n*_*I*_^*f*^(***r***) → *n*_dDFT_^*f*^(***r***), etc. Similarly, the expectation value of any operator of interest, *ô*
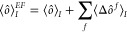
23where ⟨*Δô*^*f*^⟩_*I*_ = ⟨*ô*^*f*^⟩_dDFT_ – ⟨*ô*^*f*^⟩_*I*_. The EF approach is applicable to the forces calculation, by choosing *ô* to be the relevant operators from eq 11. For further details on the implementation of the embedded fragments method in our program, see Supporting Information, Section S3.

## 3 Statistical Analysis of sDFT Forces in the Tryptophan Zipper 2 Peptide

**Figure 1:**
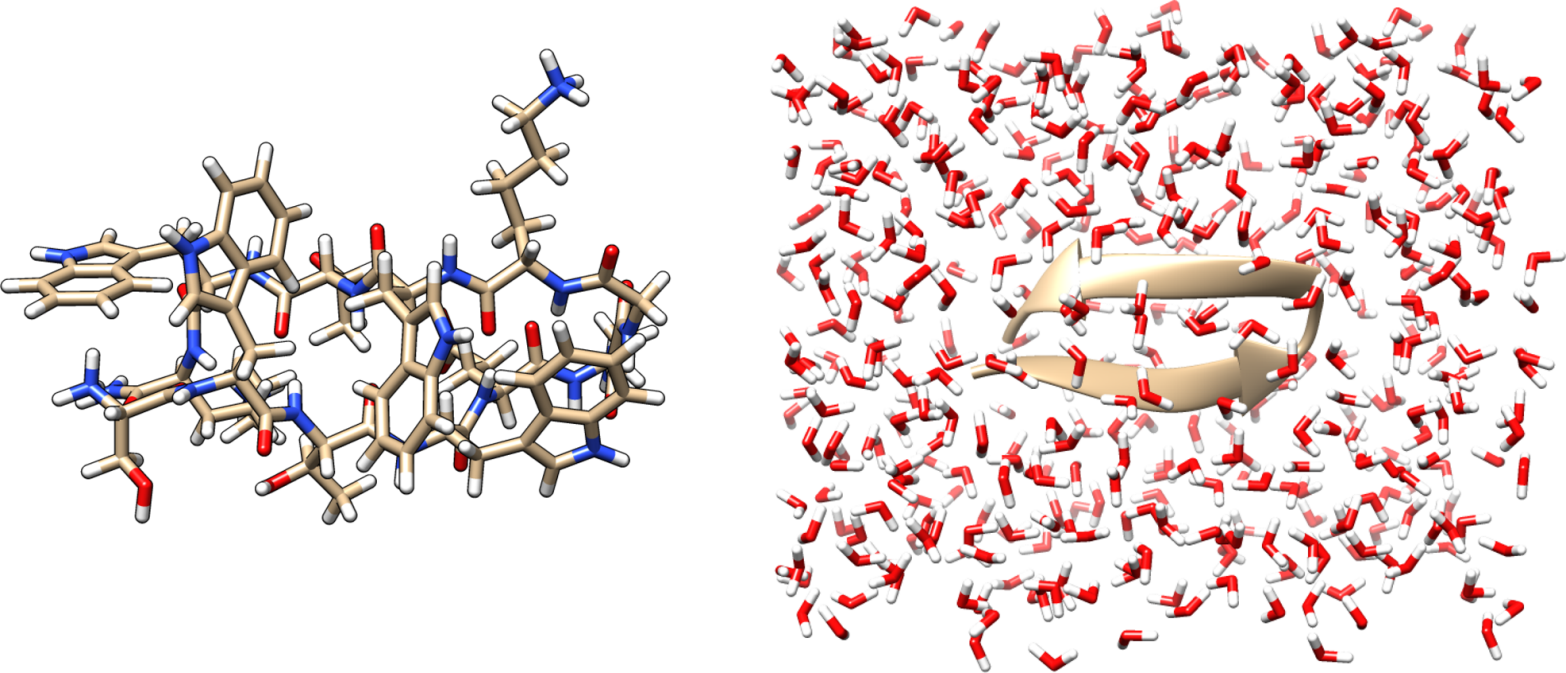
Left panel: Tryptophan Zipper 2 (Trp-zip2) peptide, composed of 220 atoms. Right panel: Trp-zip2 peptide (ribbon) solvated by 425 water molecules. The full system is composed of 1495 atoms and 4024 valence electrons, and 3118 basis functions are necessary to describe it using a minimal basis set.

Our test system is a Tryptophan Zipper 2 (Trp-zip2) peptide (pdb 1le1), composed of 220 atoms (left panel of Figure 1), solvated with 425 water molecules, and built using a universal force field (UFF) in ArgusLab^67,68^ (right panel of Figure 1). For benchmark calculations, we focused on the 20 nitrogen atoms of the peptide (indexed by *C*) and calculated the forces acting on each Cartesian degree of freedom. In these calculations, the embedded-fragment method was used, for which we chose to consider the peptide as a single fragment and then divided the 425 water molecules into 27 fragments, with an average size of 16 molecules.

To study the statistical errors, we performed the sDFT calculations using an increasing number of stochastic vectors, *I* = 12, 120, 1200, according to eq 17. To estimate the magnitudes of the bias and the uncertainty, we repeated the calculations *M* times (using independent random number generator seeds) from which we calculated a sample average force vector
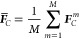
and a 3 × 3 force covariance matrix
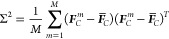
as an estimate for the covariance of the sDFT calculation. As the forces acting on each atom are represented as 3-dimensional vectors (over the Cartesian coordinates), we would like to obtain scalar values, irrespective of the way the Cartesian axes are defined, in order to estimate the uncertainty and bias of the sDFT forces. [In addition to the analysis given here, we also present the distribution of the errors *F*_*C*_^*m*^ – ***F***_*C*_^*dDFT*^ in the Supporting Information, Section S2.D.] For a canonical estimate of the uncertainty, we use an average over the eigenstates of Σ^2^
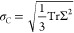
24where *F*_*C*_^*dDFT*^ = ∥***F***_*C*_^*dDFT*^∥ is the magnitude of the dDFT electronic force on atom *C*. For a canonical estimate of the bias in the force, we use the *L*_2_-Norm of the error in the average force vector:
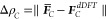
25

**Figure 2:**
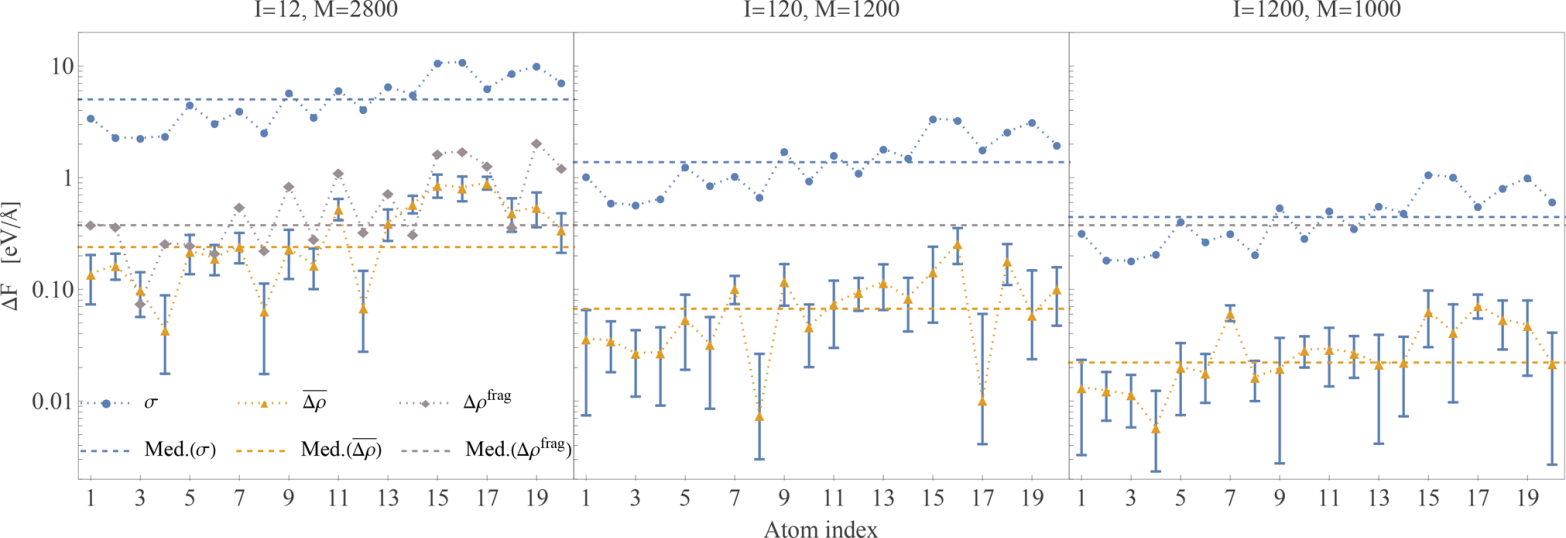
Statistical errors in the sDFT forces acting on the 20 nitrogen atoms in the solvated-TrpZip2 system calculated using *I* = 12, 120, 1200 stochastic vectors (see left, center, and right panels). For each nitrogen atom, we show the uncertainty σ_*C*_ (blue dots) and the estimate in the bias *Δρ*_*C*_ (orange triangles), see eqs 24 and 25 in the text, with error bars calculated as ±σ_*C*_/. In the *I* = 12 column, we also plot *Δρ*_*C*_^*frag*^ = ∥***F***_*C*_^*frag*^ – ***F***_*C*_^*dDFT*^∥ (gray diamonds), where ***F***_*C*_^*frag*^ is the dDFT force vector on the nitrogen atom *C* from the peptide-only fragment calculation. The dotted lines connecting the markers are presented as a guide for the eye, while the dashed horizontal lines are medians over all atoms of σ_*C*_ and *Δρ*_*C*_. For simplification of the image, in the *I* = 120, 1200 columns, we only present the median of *Δρ*_*C*_^*frag*^ (gray dashed line) taken over all 20 nitrogen atoms.

In Figure 2, we present data for the statistical errors in the forces of the 20 nitrogen atoms, ordered by an atom index according to their distance from the center of the peptide (1 closest, 20 furthest). The estimates for the uncertainty in the forces, σ_*C*_ of eq 24, are plotted in blue circles, while the estimates of the bias *Δρ*_*C*_ of eq 25, with an error bar calculated as ±σ_*C*_/, are plotted in orange triangles with blue error bars. The medians over all nitrogen atoms are plotted as dashed lines. The used number of stochastic vectors, *I*, as well as the number of repetitions, *M*, is shown above each panel. We found that stable estimates of σ_*C*_ are obtained even when using a small number of *M* ≈ 50 repetitions and observe that they obey the expected 1/ behavior in accordance with the central limit theorem. Since the variance is given by the matrix elements of the system, (see Supporting Information, Section S2.C, eq S3), the pattern seen for σ_*C*_ as a function of atom index is almost unchanged for different values of *I*. To estimate the bias, we need a good estimate of E[*m*_*I*_] (the expected value of the forces when calculated using *I* stochastic vectors in eq 18). As σ_*C*_ is much larger than *Δρ*_*C*_, a very large number of repetitions, *M*, was required in order to achieve a good enough estimate of E[*m*_*I*_] such that *Δρ*_*C*_ values are useful estimates of the bias. It is clear from the error bars that for almost all nitrogen atoms we have good estimates of the bias.

In the *I* = 12 column, for an added perspective, we plot, in gray diamonds, the error *Δρ*_*C*_^*frag*^ = ∥***F***_*C*_^*frag*^–***F***_*C*_^*dDFT*^∥, where ***F***_*C*_^*frag*^ is the force vector on the nitrogen atom *C* from a dDFT calculation on its peptide only (gas-phase) fragment. The median is given again, in a dashed line. We observe that the values of *Δρ*_*C*_^*frag*^ for the atoms closer to the center of the fragment are mostly smaller than those further away, causing a similar pattern in the sDFT errors. When comparing the median of *Δρ*_*C*_^*frag*^ (plotted for all panels in a gray dashed line) with those of the stochastic results, we see they are higher even for the *I* = 12 stochastic vectors case, whereas for the cases of *I* = 120, 1200 we observe a reduction in the errors, showing that overall sDFT significantly improves the force estimation in comparison to the deterministic fragment calculation. [We base this conclusion on the medians of *Δρ*_*C*_. The same conclusions are valid also when considering the largest error, max_*C*_{*Δρ*_*C*_}.]

Additional sDFT calculations on a smaller system, composed of the Trp-zip2 peptide and only 195 solvating water molecules, show that for a given number of stochastic orbitals (*I* = 12) the uncertainty and bias are very similar to the case of the original solvated system (see Supporting Information, Section S4). This suggests the statistical errors are roughly independent of system size.

## 4 Summary and Conclusions

We have presented a method for force calculations within finite temperature sDFT in nonorthogonal atom-centered basis sets. The forces are random variables evaluated using the stochastic trace formula applied to various operators derived from the free energy and are therefore, like all sDFT observables, characterized by statistical errors, a fluctuation, and a bias. The calculation of the forces is adapted to benefit from the embedded-fragment methodology. These calculations are dominated by the SCF sDFT convergence step, and therefore the times for force calculations are similar to those reported in ref (44).

In Section 3, we presented benchmarking calculations, focusing on the statistical errors in the force estimates for the 20 nitrogen atoms of a solvated Tryptophan Zipper 2 peptide system. The results are given as a function of *I*, the number of stochastic vectors used in the calculation according to eq 17. The uncertainty in the sDFT forces follows the expected 1/ behavior in accordance with the central limit theorem. Using a very large number of repetitions, we were also able to uncover the bias and determine that it is at least an order of magnitude smaller than the uncertainty. The magnitude of the force bias is of the order of 0.065 eV/Å (∼10^–3^*E*_*h*_/*a*_0_) when 120 stochastic orbitals are used, independently of system size. A back-of-the-envelope calculation shows that this magnitude of bias is sufficiently small to ensure that the bond lengths estimated by stochastic DFT (within a Langevin molecular dynamics simulation) will deviate by less than 1% from those predicted by a deterministic calculation. [Assuming the minimum of the Born–Oppenheimer potential is harmonic with a local force constant *k*, the bond length deviation *δR* due to a force perturbation *δF* obeys |*kδR*| = |*δF*|. In typical solids and molecules, *k* is on the order of 5 to 100 eV Å^–2^,^69,70^ so for *δF* of the order of 0.065 eV/Å, we find *δR* ≲ 0.01 Å, 1% or less for most bond lengths of interest.] Indeed, this fact was demonstrated using a Langevin Dynamics simulation on silicon nanocrystals,^42^ within a real-space representation sDFT. Our present results indicate that sDFT based on nonorthogonal atom-centered basis sets can be also used successfully in this way.

It is instructive to discuss the efficiency and accuracy of the basis set^44^ vs real-space grid^40,42^ representations of sDFT calculations. For this, we used the Si_35_H_36_ system, comparing the 6-31G basis set calculations with those of a real-space grid having 64^3^ points and grid spacing of *δx* = 0.5*a*_0_ (For more information about this comparison, see the Supporting Information, Section S5.). We find that the time for application of the density matrix to a random vector in the 6-31G basis is a factor of 30 faster than in the grid representation. On the other hand, surprisingly, the standard deviation of fluctuations in a typical Si force component is about 5 times larger in the basis set calculation than in the grid. Therefore, we need a factor of 5^2^ = 25 more stochastic vectors (because their number is proportional to the square of the standard deviation) in the basis set calculation for achieving the same fluctuation error. If we had only a single processor, the two representations would thus require a similar numerical effort for achieving a given fluctuation goal: the grid is 30 times slower but requires a factor of 25 less samplings. Due to the highly parallelizable nature of sDFT, the necessary extra sampling required by the basis-set-based calculation does not automatically lead to increased wall-times, if additional CPUs can be offered. We conclude that the basis-set-based calculations can achieve smaller wall-times than real-space grids, given additional CPUs.

## Appendix A: Derivation of the Changes in Free Energy

Here, we derive the force expression of eq 11. The force is given by the change in free energy

due to displacement of the nuclei. When nuclei are displaced, the DM also changes, and we will show that under any change in the density matrix *P* → *P* + δ_0_*P*, while keeping the nuclei fixed, the free energy of eq 8 does not change when *P* is given by eq 3. This will be done by examining each term in the above equation separately and summing over all of them. Then, we will consider the direct change in free energy due to a displacement of the nuclei (while *P* is held constant). It is only this latter change which affects the free energy.

### A.1 The Variation in

Starting from

and

Combining these we see

We consider two types of variations: δ_0_ which changes the DM but not the atoms and δ_*C*_ which changes the position of atom *C* (and thus affects the basis functions associated with that atom) but not *P*.

1. *P* → *P* + δ_0_*P* (assuming nuclei are constant): Here
  26
  so
  27
2. Nucleus C moves by δ_*C*_*X* (and ϕ_α_ → ϕ_α_ + δ_*C*_ϕ_α_) (constraining *P* to be constant): the change in the density is
  so
  28
  using the change in the overlap matrix
  29

### A.2 The Variation in

Starting from

we have two types of variations: δ_0_ which changes the DM but not the atoms and δ_*C*_ which changes the position of atom *C* (and thus affects the basis functions associated with that atom) but not *P*.

1. *P* → *P* + δ_0_*P* (freezing the nuclei). We have that
  so using eq 26
  30
2. Nucleus C moves by δ_*C*_*X* (and ϕ_α_→ ϕ_α_ + δ_*C*_ϕ_α_) (constraining *P* to be constant): we find
  31
  where

32

### A.3 The Variation in

Starting from  = −2 × *k*_B_Tr[*SP* ln (*SP*) + (1 – *SP*) ln (1 – *SP*)],

1. *P* → *P* + δ_0_*P* (freezing the nuclei). We have by derivation that
  33
2. Nucleus C moves by δ_*C*_*X* (and ϕ_α_ → ϕ_α_ + δ_*C*_ϕ_α_) (constraining *P* to be constant): we find

34

### A.4 The Variation in Ω[*P*]

Here, we combine the above results, while using the relationship

which we find by substituting in eq 3 for *P*.

1. *P* → *P* + δ_0_*P* (freezing the nuclei). Using eqs 27, 30, and 33, we have
  leading to
  This reflects the fact that *P* of eq 3 minimizes Ω [*P*].
2. Nucleus C moves by δ_*C*_*X* (and ϕ_α_ → ϕ_α_ + δ_*C*_ϕ_α_) (since a variation in *P* does not affect the value of Ω, we can take it as a constant): using eqs 28, 31, and 34, we find
  35
  The change in free energy is composed of two terms: a term due to the energy, δ_*C*_*H* (which includes a direct change and a Pulay term, see eq 32), and a change due to entropy, which depends purely on Pulay changes in the overlap matrix, δ_*C*_*S* (see (29)).

## Supporting Information Available

Evaluation of force matrix elements (Section S1), basic concepts in statistics (Section S2), embedded fragment method (Section S3), system size dependency of statistical errors (Section S4), and efficiency of representations (Section S5) (PDF)
