# Supplementary material for: Forces from Stochastic Density Functional Theory under Nonorthogonal Atom-Centered Basis Sets
Source: J Chem Theory Comput. 2022 Jan 31;18(3):1458–66. doi: 10.1021/acs.jctc.1c00794 (PMC8908760; doi:10.1021/acs.jctc.1c00794)
Supplement: Supplementary file 1 — ct1c00794_si_001.pdf [file ct1c00794_si_001.pdf]

Supporting Information:
Forces from Stochastic Density Functional Theory under Nonorthogonal Atom-Centered Basis Sets

Ben Shpiro
Fritz Haber Center for Molecular Dynamics and Institute of Chemistry, The Hebrew University of Jerusalem, Jerusalem 9190401, Israel

Marcel David Fabian
Fritz Haber Center for Molecular Dynamics and Institute of Chemistry, The Hebrew University of Jerusalem, Jerusalem 9190401, Israel

Eran Rabani*
Department of Chemistry, University of California, Berkeley, California 94720, United States Materials Sciences Division, Lawrence Berkeley National Laboratory, Berkeley, California 94720, United States and The Raymond and Beverly Sackler Center of Computational Molecular and Materials Science, Tel Aviv University, Tel Aviv 69978, Israel

Roi Baer†
Fritz Haber Center for Molecular Dynamics and Institute of Chemistry, The Hebrew University of Jerusalem, Jerusalem 9190401, Israel

S1. EVALUATING MATRIX ELEMENTS FOR FORCES

In this section we will describe how to calculate $(\delta_C S)_{\alpha\beta} = \left(\frac{\partial}{\partial X_C} S\right)_{\alpha\beta} \delta_C X$:

$$\left(\frac{\partial}{\partial X_C} S\right)_{\alpha\beta} = \left\langle \frac{\partial}{\partial X_C}\phi_\alpha \middle| \phi_\beta \right\rangle + \left\langle \phi_\alpha \middle| \frac{\partial}{\partial X_C}\phi_\beta \right\rangle$$

and $(\delta_C H)_{\alpha\beta} = \left(\frac{\partial}{\partial X_C} H\right)_{\alpha\beta} \delta_C X$:

$$\left(\frac{\partial}{\partial X_C} H\right)_{\alpha\beta} = \left\langle \phi_\alpha \middle| \frac{\partial}{\partial X_C}\hat{h}_{KS} \middle| \phi_\beta \right\rangle + \left\langle \frac{\partial}{\partial X_C}\phi_\alpha \middle| \hat{h}_{KS} \middle| \phi_\beta \right\rangle + \left\langle \phi_\alpha \middle| \hat{h}_{KS} \middle| \frac{\partial}{\partial X_C}\phi_\beta \right\rangle$$

used in Eq. 11 in the manuscript.

A. The Pulay forces

Here we give the detail of calculating the $\left\langle \frac{\partial}{\partial X_C}\phi_\alpha \middle| \phi_\beta \right\rangle$ and $\left\langle \frac{\partial}{\partial X_C}\phi_\alpha \middle| \hat{h}_{KS} \middle| \phi_\beta \right\rangle$ type matrix elements. For these we need to calculate the derivatives of the basis functions $\phi(\boldsymbol{r}_g - \boldsymbol{R}_C)$ with respect to nuclear coordinates $R_C = (X_C, Y_C, Z_C)$. Since we use Cartesian Gaussian functions as our basis we enjoy the ease of calculating their values and derivatives analytically on the grid. Each atom-centered basis function is a sum of primitives, $e^{-\gamma x^2} x^l \times e^{-\gamma y^2} y^m \times e^{-\gamma x^2} z^n$, where $n+l+m$ is the total angular momentum quantum number. Therefore each primitive can be represented by three 1-dimensional vectors $\xi_\alpha(x_g - X_C)$, $\eta_\alpha(y_g - Y_C)$ and $\zeta_\alpha(z_g - Z_C)$ and the function is defined inside a "window" surrounding the atom C and given as a product of three terms at each of its grid points:

$$\phi_\alpha(\boldsymbol{r}_g) = \xi_\alpha(x_g - X_C)\,\eta_\alpha(y_g - Y_C)\,\zeta_\alpha(z_g - Z_C)\,.$$

---

* eran.rabani@berkeley.edu
† roi.baer@huji.ac.il

The grid is then used to evaluate the matrix elements of the overlap and Hamiltonian matrices:

$$S_{\alpha\beta} = h^3 \sum_{\boldsymbol{r}_g} \phi_\alpha(\boldsymbol{r}_g)\, \phi_\beta(\boldsymbol{r}_g)\,,$$

$$H_{\alpha\beta} = h^3 \sum_{\boldsymbol{r}_g} \phi_\alpha(\boldsymbol{r}_g) \left[\hat{h}_{KS}\phi_\beta\right](\boldsymbol{r}_g)\,,$$

where h is the uniform grid-spacing.

Since the forces are the derivatives with respect to nuclear coordinate X_C, while our grid points are the electronic coordinates, x_g, we use the fact that for a basis function centered around $\boldsymbol{R}_C$, taking the derivative with respect to X_C is simply the negative of the derivative with respect to x evaluated at $\boldsymbol{r}_g - \boldsymbol{R}_C$:

$$\frac{\partial}{\partial X_C}\phi_\alpha(\boldsymbol{r}_g) = -\xi'_\alpha(x_g - X_C)\,\eta_\alpha(y_g - Y_C)\,\zeta_\alpha(z_g - Z_C)\,,$$

such that derivatives can therefore also be defined inside a "window" surrounding the atom C and given as a product of three terms at each of its grid points. The grid is then used to evaluate the Pulay matrix elements of $\delta_C S$ and $\delta_C H$:

$$\left\langle \frac{\partial}{\partial X_C}\phi_\alpha \middle| \phi_\beta \right\rangle = h^3 \sum_{\boldsymbol{r}_g} \left[\frac{\partial}{\partial X_C}\phi_\alpha(\boldsymbol{r}_g)\right] \phi_\beta(\boldsymbol{r}_g)\,,$$

$$\left\langle \frac{\partial}{\partial X_C}\phi_\alpha \middle| \hat{h}_{KS} \middle| \phi_\beta \right\rangle = h^3 \sum_{\boldsymbol{r}_g} \left[\frac{\partial}{\partial X_C}\phi_\alpha(\boldsymbol{r}_g)\right] \left[\hat{h}_{KS}\phi_\beta\right](\boldsymbol{r}_g)\,,$$

The above Pulay terms are non-zero only when the ϕ_α basis belongs to atom C, i.e. when $\phi_\alpha \in C$, and therefore need to be calculated only for a very small number of α, β pairs, i.e. only when ϕ_α, ϕ_β have overlapping windows on the grid. The result of these conditions is that Pulay force matrices are incredibly sparse and their number of non-zero elements, $N_{\text{non zero}}$, is independent of the system size, as it depends only on the choice of basis set through the number of basis functions per atom. As for each degree of freedom (DOF), X_C (X direction of atom C), we have to compute a Pulay force sparse-matrix, we exploit these conditions in our code by only evaluating non-zero matrix elements and storing them in sparse-matrix structures. In Subsection S1 C we describe the method of storage and application (onto a vector) of the sparse structure we have used.

The algorithm for computing $\left(\frac{\partial}{\partial X_C} S\right)_{\alpha\beta}$

- For all basis functions $\alpha \in C$
  - loop over all basis function $\beta < \alpha$ that overlap with α, then:
  - If $\beta \in C$

$$\left(\frac{\partial S}{\partial X_C}\right)_{\alpha\beta} = \left\langle \frac{\partial}{\partial X_C}\phi_\alpha \middle| \phi_\beta \right\rangle + \left\langle \phi_\alpha \middle| \frac{\partial}{\partial X_C}\phi_\beta \right\rangle$$

  - otherwise

$$\left(\frac{\partial S}{\partial X_C}\right)_{\alpha\beta} = \left\langle \frac{\partial}{\partial X_C}\phi_\alpha \middle| \phi_\beta \right\rangle$$

- For all basis functions $\alpha \notin C$
  - loop over all basis function $\beta < \alpha$ that overlap with α, then:
  - If $\beta \in C$

$$\left(\frac{\partial S}{\partial X_C}\right)_{\alpha\beta} = \left\langle \phi_\alpha \middle| \frac{\partial}{\partial X_C}\phi_\beta \right\rangle$$

- All other terms are not 0, and we do not store them in the sparse matrix structure.

(To simplify the code, as $\left\langle \phi_\alpha \middle| \frac{\partial}{\partial X_C}\phi_\beta \right\rangle = \left\langle \frac{\partial}{\partial X_C}\phi_\beta \middle| \phi_\alpha \right\rangle$, we always take the derivative from the left side).

Overall we get:

$$\left(\frac{\partial S}{\partial X_C}\right)_{\alpha\beta} = \begin{cases} \left\langle \frac{\partial}{\partial X_C}\phi_\alpha \middle| \phi_\beta \right\rangle + \left\langle \phi_\alpha \middle| \frac{\partial}{\partial X_C}\phi_\beta \right\rangle & \alpha,\beta \in C \\ \left\langle \frac{\partial}{\partial X_C}\phi_\alpha \middle| \phi_\beta \right\rangle & \alpha \in C, \beta \notin C \\ \left\langle \phi_\alpha \middle| \frac{\partial}{\partial X_C}\phi_\beta \right\rangle & \alpha \notin C, \beta \in C \\ 0 & \alpha,\beta \notin C \end{cases}$$

and the same can be done for the Hamiltonian Pulay terms:

$$\left(\frac{\partial H}{\partial X_C}\right)^{\text{Pulay}}_{\alpha\beta} = \begin{cases} \left\langle \frac{\partial}{\partial X_C}\phi_\alpha \middle| \hat{h}_{KS} \middle| \phi_\beta \right\rangle + \left\langle \phi_\alpha \middle| \hat{h}_{KS} \middle| \frac{\partial}{\partial X_C}\phi_\beta \right\rangle & \alpha,\beta \in C \\ \left\langle \frac{\partial}{\partial X_C}\phi_\alpha \middle| \hat{h}_{KS} \middle| \phi_\beta \right\rangle & \alpha \in C, \beta \notin C \\ \left\langle \phi_\alpha \middle| \hat{h}_{KS} \middle| \frac{\partial}{\partial X_C}\phi_\beta \right\rangle & \alpha \notin C, \beta \in C \\ 0 & \alpha,\beta \notin C \end{cases}$$

however, as the Hamiltonian includes terms that are explicitly dependent on nuclear coordinates in the form of the non-local (nl) and local (loc) pseudopotential terms: $\hat{v}_{pp}^{nl/loc} = \sum_{C' \in nuclei} \hat{v}_{pp(C')}^{nl/loc}$, there are also $\left\langle \phi_\alpha \middle| \frac{\partial}{\partial X_C}\hat{h}_{KS} \middle| \phi_\beta \right\rangle = \left\langle \phi_\alpha \middle| \frac{\partial}{\partial X_C}\hat{v}_{pp}^{nl/loc} \middle| \phi_\beta \right\rangle$ type terms that contribute to the overall force on atom C in the X direction. See Subsection S1 B below for the detail of these force terms.

B. The direct forces

Here we give the detail of calculating the $\left\langle \phi_\alpha \middle| \frac{\partial}{\partial X_C}\hat{v}_{pp}^{nl/loc} \middle| \phi_\beta \right\rangle$ type matrix elements.

Since the non-local and local pseudopotential operators are $\hat{v}_{pp}^{nl/loc} = \sum_{C' \in nuclei} \hat{v}_{pp(C')}^{nl/loc}$, the derivative with respect to a variation in nuclear coordinate $X_C$is given by:

$$\frac{\partial}{\partial X_C}\hat{v}_{pp}^{nl/loc} \left|\phi_\beta\right\rangle = \frac{\partial}{\partial X_C}\hat{v}_{pp(C)}^{nl/loc} \left|\phi_\beta\right\rangle .$$

The $\hat{v}_{pp(C)}^{nl/loc}$ operators have an analytical expression of the Kleinman-Bylander form [1], such that we can apply it, and its derivative, $\frac{\partial}{\partial X_C}\hat{v}_{pp(C)}^{nl/loc}$, on a vector on the grid.

Due to its short-range nature, $\hat{v}_{pp(C)}^{nl}$ and subsequently $\frac{\partial}{\partial X_C}\hat{v}_{pp(C)}^{nl}$ are stored on a small "window" of grid points around R_C. The $\left\langle \phi_\alpha \middle| \frac{\partial}{\partial X_C}\hat{v}_{pp(C)}^{nl} \middle| \phi_\beta \right\rangle$ matrix elements are therefore calculated as a multiplication of two grid vectors:

$$\left(\frac{\partial}{\partial X_C}V_{pp(C)}^{nl}\right)^{\text{direct}}_{\alpha\beta} = h^3 \sum_{\boldsymbol{r}_g \in \left(\alpha \cap \beta \cap \hat{v}_{pp(C)}^{nl}\right)} \phi_\alpha\left(\boldsymbol{r_g}\right) \left[\left[\frac{\partial}{\partial X_C}\hat{v}_{pp(C)}^{nl}\right]\phi_\beta\right]\left(\boldsymbol{r_g}\right), \tag{S1}$$

where the sum over grid points $\boldsymbol{r_g}$ is over only grid points that are inside the windows of all three terms, ϕ_α, $\phi_\beta$and $\hat{v}_{pp(C)}^{nl}$. This requirement of overlapping windows for all three terms results in very sparse matrices, with the number of non-zero matrix elements, $N_{\text{non zeros}}$, dependent only on the choice of basis set and independent of system size.

For each degree of freedom (DOF), X_C (atom C in the X direction), we have to compute a $\frac{\partial}{\partial X_C}V_{pp(C)}^{nl}$ force sparse-matrix and apply it to a stochastic vector as part of the evaluation using the stochastic trace formula. In Subsection S1 C we describe the method of storage and application of the sparse structure we have used.

The algorithm for computing $\left(\frac{\partial}{\partial X_C}V^{nl}_{pp(C)}\right)^{direct}_{\alpha\beta}$

- loop over all basis functions β that have overlapping windows with $\hat{v}^{nl}_{pp(C)}$
  - Calculate the $\left[\frac{\partial}{\partial X_C}\hat{v}^{nl}_{pp(C)}\phi_\beta\right](\boldsymbol{r}_g)$ grid vector for all $\boldsymbol{r}_g \in \beta \cap \hat{v}^{nl}_{pp(C)}$
    - ∗ loop over all basis functions α that overlap with both β, $\hat{v}^{nl}_{pp(C)}$
      - · Calculate $\left(\frac{\partial}{\partial X_C}V^{nl}_{pp(C)}\right)_{\alpha\beta}$ according to Eq.(S1)

The local PP operator, $\hat{v}^{loc}_{pp}$, (which includes the long range Coulomb attraction), depends directly on the density $n(\boldsymbol{r}_g)$. In this case, an equivalent method to the trace calculation of the direct $\frac{\partial}{\partial X_C}\hat{v}^{loc}_{pp}$ is done by usual planewaves calculations on the grid using $n(\boldsymbol{r}_g)$ in reciprocal space. This scaling of this approach is quadratic with system size when the forces on all atoms are calculated, however it is highly efficient.

C. Sparse matrix structures for Pulay and non-local PP forces

To exploit the sparsity of the above, direct and Pulay, force matrices, we do not store them in full $K \times K$ matrix structures (where K is the number of basis functions), but rather use compact a storage structure. In these, for every degree of freedom we store three lists of length $N_{\text{non zeros}}$: (val,I,J) $\equiv M_{ij}$ where (I,J) gives the location of the value, val, in the $K \times K$ matrix M.

$$M = \begin{pmatrix} 0 & \cdots & 0 & 0 \\ \vdots & \ddots & \vdots & \vdots \\ 0 & \cdots & a & b \\ 0 & \cdots & c & d \end{pmatrix}_{K\times K} \equiv \overset{\text{val}}{\begin{pmatrix} a \\ b \\ c \\ d \end{pmatrix}} \overset{\text{I}}{\begin{pmatrix} K-1 \\ K-1 \\ K \\ K \end{pmatrix}} \overset{\text{J}}{\begin{pmatrix} K-1 \\ K \\ K-1 \\ K \end{pmatrix}}$$

As per the above example, the sparse structure allows for a significant reduction in the number of stored values in memory as we only store $(3 \times N_{\text{non zeros}})$ elements per DOF as opposed to K^2 elements per DOF. Since the number of DOF's, $N_{\text{DOF}} \propto K$ and since $N_{\text{non zeros}}$ is a small number dependent only on the choice of basis set (but not on system size!) our sparse structure reduces the scaling of the memory requirement from $\mathcal{O}(K^3)$ to $\mathcal{O}(K)$.

The sparse structure also allows for an efficient matrix vector multiplication. For a matrix M and vector $|\boldsymbol{z}\rangle$ the operation

$$|\boldsymbol{y}\rangle = M\,|\boldsymbol{z}\rangle$$

is given by:

$$y_k = \sum_{n,\, I(n)=k} \text{val}(n) \times z_{\text{J}(n)}$$

such that we only require $N_{\text{non zeros}}$ multiplications for a matrix vector operation (as all terms of c, that are not in the I list, are zero).

For the stochastic trace formula we need to calculate expectation values using the stochastic vectors, χ. In a bra-ket notation, for a matrix M:

$$r = \langle\chi\,|M|\,\chi\rangle$$

is given by:

$$r = \sum_{n}^{N_{\text{non zeros}}} \chi_{\text{I}(n)} \times \text{val}(n) \times \chi_{\text{J}(n)}$$

such that we only require $2 \times N_{\text{non zeros}}$ multiplications. Since the calculation of the force operators' expectation values (direct and Pulay), per DOF, are independent of system size, the overall scaling of the force calculations using this sparse structure is $\mathcal{O}(K)$.

S2. BASIC CONCEPTS IN STATISTICS

A. Random variables

In order to understand the statistical errors involved in our procedures we briefly review the concept of a random variable r [2]. It takes any one of a discrete set of values $\{r\}$ with a given probability $p(r) \geq 0$, where $\sum_r p(r) = 1$. The expected value of r is: $\mathrm{E}[r] = \sum_r rp(r)$ and the variance is $\mathrm{Var}[r] = \mathrm{E}\left[(r - \mathrm{E}[r])^2\right]$. Using a sample of I independent draws from the population of r's we calculate the mean

$$m_I = \frac{1}{I}\sum_{i=1}^{I} r_i$$

and the standard deviation

$$s_I = \sqrt{\frac{1}{I-1}\sum_{i=1}^{I}(r_i - m_I)^2},$$

both can also be viewed as random variables with appropriate probability functions themselves. It can be shown that

1. The expected value of m_I is the same as that of r:

$$\mathrm{E}[m_I] = \mathrm{E}[r]$$

2. The variance of m_I is smaller by a factor I than that of r:

$$\mathrm{Var}[m_I] = \frac{1}{I}\mathrm{Var}[r]$$

3. The expected value of s_I^2 is equal to the variance of:

$$\mathrm{E}\left[s_I^2\right] = \mathrm{Var}[r].$$

From these properties, m_I and s_I^2 can serve as unbiased estimators of the expected value and variance of the original random variable. When I is sufficiently large, the interval of values $[m_I - \sigma_I, m_I + \sigma_I]$, where

$$\sigma_I = \frac{s_I}{\sqrt{I}}$$

is the uncertainty giving a 70% confidence interval for $\mathrm{E}[r]$. Based on the sampled data, there is a probability of 70% that $E[r]$ falls within this interval.

B. Stochastic vectors

In section 2.4 of the main text we discuss the stochastic evaluation of observables using the stochastic trace formula

$$\mathrm{Tr}[A] = \mathrm{E}\left[\chi^T A \chi\right], \tag{S2}$$

where we treat each $\chi^T A \chi$ as a random variable. The variance associated the result is given by

$$\mathrm{Var}\left[\chi^T A \chi\right] = \frac{1}{2}\sum_{i \neq j}(A_{ij} + A_{ji})^2. \tag{S3}$$

The relation in Eq. (S2) is called the stochastic trace formula and it allows evaluating the trace of A by parameter estimation techniques based on statistical sampling theory.

Proof of Eq. (S3) We begin the proof using the definition of the variance of a random variable, $\mathrm{Var}[x] = \mathrm{E}[x^2] - \mathrm{E}[x]^2$, and considering $\chi^T A \chi$ as our random variable:

$$\begin{aligned}\mathrm{Var}\left[\chi^T A\chi\right] &= \mathrm{E}\left[\left(\chi^T A\chi\right)^T \chi^T A\chi\right] - \left(\mathrm{E}\left[\chi^T A\chi\right]\right)^2 \\ &= \mathrm{E}\left[\chi^T A^T \chi\chi^T A\chi\right] - \left(\mathrm{Tr}\left[A\right]\right)^2 \\ &= \mathrm{E}\left[\chi_k\chi_l\chi_i\chi_j\right] A_{kl}A_{ij} - \left(\mathrm{Tr}\left[A\right]\right)^2 \end{aligned} \tag{S4}$$

where in the second line we have used Eq. (S2), and in the third line the fact that the matrix A is completely deterministic. We will now evaluate $\mathrm{E}[\chi_k\chi_l\chi_i\chi_j]$, using that $\mathrm{E}[\chi_i\chi_j] = \delta_{ij}$:

$$\begin{aligned}\mathrm{E}\left[\chi_k\chi_l\chi_i\chi_j\right] &= \delta_{kl}\mathrm{E}\left[\chi_i\chi_j\right] + (1-\delta_{kl})\left(\delta_{ki}\mathrm{E}\left[\chi_l\chi_j\right] + (1-\delta_{ki})\left(\delta_{kj}\mathrm{E}\left[\chi_i\chi_l\right]\right)\right) \\ &= \delta_{kl}\delta_{ij} + (1-\delta_{kl})\left(\delta_{ki}\delta_{lj} + (1-\delta_{ki})\,\delta_{kj}\delta_{il}\right) \\ &= \delta_{kl}\delta_{ij} + \delta_{ki}\delta_{lj} + \delta_{kj}\delta_{il} - 2\delta_{kl}\delta_{kj}\delta_{ik}\end{aligned}$$

and multiply by $A_{kl}A_{ij}$ and sum over all indices:

$$\begin{aligned}\mathrm{E}\left[\chi_k\chi_l\chi_i\chi_j\right] A_{kl}A_{ij} &= A_{kk}A_{ii} + A_{ij}A_{ij} + A_{ji}A_{ij} - 2A_{ii}^2 \\ &= \mathrm{Tr}\left[A\right]^2 + \sum_{i\neq j} A_{ij}\left(A_{ij} + A_{ji}\right) \\ &= \mathrm{Tr}\left[A\right]^2 + \frac{1}{2}\sum_{i\neq j}\left(A_{ij} + A_{ji}\right)^2\end{aligned}$$

Substituting back into Eq. (S4), we arrive at

$$\mathrm{Var}\left[\chi^T A\chi\right] = \frac{1}{2}\sum_{k\neq l}\left(A_{kl} + A_{lk}\right)^2$$

C. Parameter estimation and statistical errors

Often expected value $\mathrm{E}[r]$ of a distribution of a random variable r is not known. The estimation of this parameter can be done, based on the use of a finite sample r_i of size I, as discussed in section S2 A. As an demonstration of this procedure we return to the question of how to evaluate the $\mathrm{Tr}[A]$. We take a sample of I stochastic vectors χ_i and form a random variable $\frac{1}{I}\sum_{i=1}^{I}\chi_i^T A\chi_i$. Then

$$\mathrm{Tr}\left[A\right] = \mathrm{E}\left[\frac{1}{I}\sum_{i=1}^{I}\chi_i^T A\chi_i\right], \tag{S5}$$

and

$$\mathrm{Var}\left[\frac{1}{I}\sum_{i=1}^{I}\chi_i^T A\chi_i\right] = \frac{1}{2I}\sum_{k\neq l}\left(A_{kl} + A_{lk}\right)^2.$$

As discussed above, the sample mean $m_I = \frac{1}{I}\sum_{i=1}^{I}\chi_i^T A\chi_i$ and corresponding standard deviation s_I can be used to provide a confidence interval of uncertainty $\sigma_I = s_I/\sqrt{I}$ for the value of $\mathrm{Tr}[A]$. This statistical approach, of building a confidence interval for $\mathrm{Tr}[A]$ involves I applications of A to a vector, whereas the deterministic calculation of $\mathrm{Tr}[A]$ involves K such applications. Therefore, as long as $I \ll K$ we obtain a large saving in the numerical effort, but at the price of introducing an uncertainty.

Now, suppose we wanted to estimate a given function of the expected value of a random variable, $f(\mathrm{E}[r])$. The simplest procedure is apply f to the sample mean m_I and take $f(m_I)$ as such an estimate. This procedure works when $f(x)$ is a linear function of x but otherwise will generally incur a systematic error, called a bias. For example, when $f(x) = x^2$ and r is a random variable with $\mathrm{E}[r] = 0$ and $\mathrm{Var}[r] = 1$, then $\mathrm{E}[f(m_I)] = \mathrm{E}\left[m_I^2\right] = \frac{\mathrm{E}[r^2]}{I} = \frac{1}{I}$ which is clearly different from the exact value of $f[\mathrm{E}[r]] = \mathrm{E}[r]^2 = 0$. Hence we have the undesirable case, that for a finite value of I, errors will involve fluctuations around the wrong value. Note however, that as I grows, the bias diminishes in proportion to $\frac{1}{I}$. To be useful, when a bias exists, we need to make sure it is of sufficiently small magnitude.

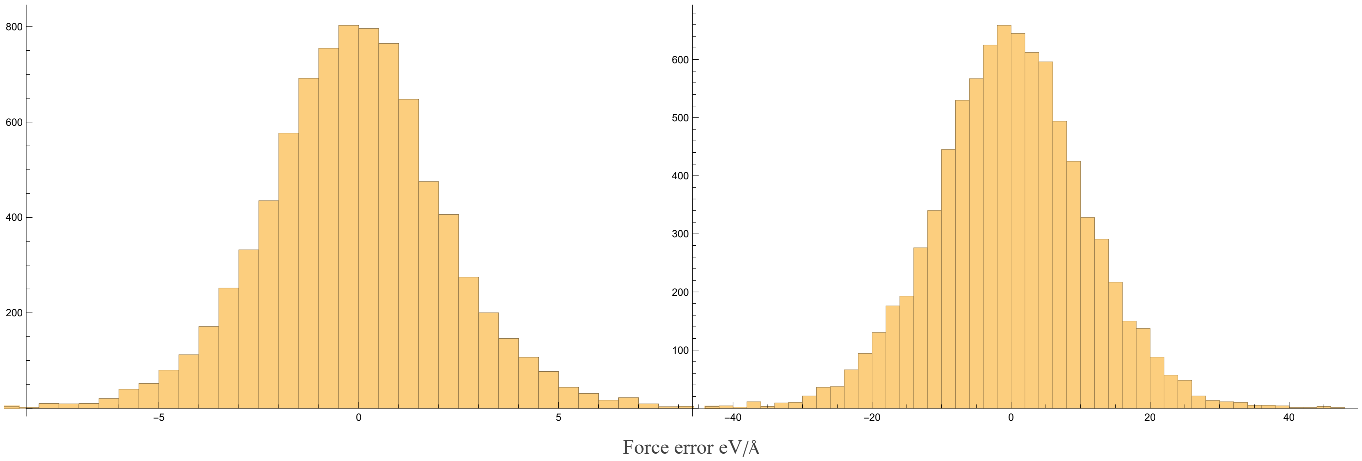

Figure S1. Histograms representing the distribution in the errors in the sDFT forces with $I = 12$ stochastic vectors. We show the errors of forces acting on two nitrogen atoms from the solvated-TrpZip2 system. On the left (right) panel is the data for the smallest (largest) standard deviation cases from all nitrogen atoms. The data for the histogram was collected by repeating the sDFT force calculation $M = 2800$ times and comparing the force vector $\boldsymbol{F}_C^m$ on each atom C to the corresponding deterministic (accurate) value $\boldsymbol{F}_C^{dDFT}$. Thus we obtained $3 \times M$ error values in the x-y-z components of the force, which represent the scatter of force components for the atom.

D. Distribution of random errors in the forces

In Fig. S1 we present histograms of the sDFT force errors $(\boldsymbol{F}_C^m - \boldsymbol{F}_C^{dDFT},\ m = 1, \ldots, M)$ on two nitrogen atoms in the solvated-TrpZip2 system. We selected the atoms that have the smallest/largest standard deviation. For both atoms, we find a Gaussian-looking distribution of the force errors centered around zero with a standard deviation within the bounds reported in the Manuscript.

S3. EMBEDDED FRAGMENTS

A. Calculation detail in the sDFT code

We would like to explain here how the equation:

$$\langle \hat{o} \rangle_I^{EF} = \langle \hat{o} \rangle_I + \sum_f \left\langle \Delta \hat{o}^f \right\rangle_I \tag{S6}$$

is implemented within our sDFT code. We give here the steps in the calculation of the correction terms from each fragment,$\left\langle \Delta \hat{o}^f \right\rangle_I$, where we defined the fragment-based correction as the difference between the dDFT and sDFT, for every fragment:

$$\left\langle \Delta \hat{o}^f \right\rangle_I = \left\langle \hat{o}^f \right\rangle_{dDFT} - \left\langle \hat{o}^f \right\rangle_I$$

For the calculation of $\left\langle \hat{O} \right\rangle_{\text{dDFT}}^f$ we solve the generalised eigenvalue problem, find P^f, the density matrix of the fragment subsystem, and trace:

$$\langle \hat{o} \rangle_{\text{dDFT}}^f = 2\ \text{Tr}\left[(OP)^f \right] \tag{S7}$$

while $\langle \hat{o} \rangle_I^f$ is calculated using the stochastic trace formula:

$$\langle \hat{o} \rangle_I^f = 2\, \frac{1}{I} \sum_{i=1}^{I} \left(\chi_i^T \right)^f (OP)^f \chi_i^f$$

with the vectors χ_i^f that are "cut-outs" of the vectors χ_i which we use in the stochastic trace formula for the full system, such that the stochastic element in the fragment calculation corresponds with the one made on the full system. Rearranging the above equation to be written as a trace expression gives:

$$\langle \hat{o} \rangle_I^f = 2\,\mathrm{Tr}\left[(OP)^f \left(\frac{1}{I} \sum_{i=1}^{I} \chi_i^f \left(\chi_i^T\right)^f \right) \right]$$

Finally, for the deterministic trace of Eq. (S7) we multiply $(OP)^f$ by $(\mathrm{Id})^f$, the identity matrix of the fragment dimensions, which allows us to rewrite the fragment correction as:

$$\left\langle \Delta \hat{o}^f \right\rangle_I = 2\,\mathrm{Tr}\left[(OP\Delta_I)^f \right]$$

where $\Delta_I^f = (\mathrm{Id})^f - \frac{1}{I}\sum_{i=1}^{I} \chi_i^f \left(\chi_i^T\right)^f$.

B. Another look at the fragments

A second useful outlook on the embedded-fragments method gives insight into the reason the statistical errors are reduced when the method is used. We rearrange Eq. (S6) to get:

$$\begin{aligned} \langle \hat{o} \rangle_I^{EF} &= \sum_f \langle \hat{o} \rangle_{\mathrm{dDFT}}^f + \left[\langle \hat{o} \rangle_I - \sum_f \langle \hat{o} \rangle_I^f \right] \\ &= 2\,\mathrm{Tr}^{det}\left[\sum_f (OP)^f \right] + 2\,\mathrm{Tr}^{stoch}\left[O\left(P - \sum_f P^f \right) \right] \end{aligned} \tag{S8}$$

such that$\mathrm{Tr}^{stoch}\left[O\left(P - \sum_f P^f \right) \right]$ is a stochastic correction to the dDFT calculation summed over all fragments. As per Eq. (S3) the variance in the stochastic trace is given by the magnitude of the off-diagonal matrix elements of $O\left(P - \sum_f P^f \right)$, and so clearly, as $\sum_f P^f$ approaches P the stochastic trace will have much smaller variance.

S4. SYSTEM SIZE DEPENDENCY

We compare the statistical errors of sDFT forces of two Trp-zip2 peptide systems, each with a different number of solvating water molecules. The first is the system we presented results for in Section III of the manuscript, solvated by 425 water molecules, while the second is a smaller system, with only 195 solvating water molecules. To keep the systems as similar as possible we cropped the smaller system directly from the larger one, such that the 195 solvating water molecules that are closest to the peptide are identical in their geometry in both systems, and the embedded-fragment method was employed such that, for both systems, the entire peptide composes one fragment, while the remaining water molecules are split into fragments with an average number of 16 water molecules per fragment.

In Fig. S2 we present the uncertainty and bias estimates for the sDFT forces on the 20 nitrogen atoms of the peptide, in the two systems described above, for the case of $I = 12$ stochastic orbitals. To allow for a comparison of the bias estimates, we repeated the calculation a large, $M = 2800$, times. The uncertainty values of each nitrogen atom are near identical between the two systems. Since the errors fluctuate, even for 2800 repetitions, we compare the median value over all nitrogen atoms, and find that it is practically identical for the two systems. These results give indication to a weak dependency of the statistical errors, uncertainty and bias, on system size.

S5. COMPARISON WITH REAL-SPACE GRID REPRESENTATION

We have checked the speed of basis sets vs. real-space grid calculations (as described in Ref. [3]) with $Si_{35}H_{36}$ as a benchmark. For the real-space grid calculations we used a grid of 64^3 points of spacing $\delta x = 0.5a_0$ and the wall time for the real-space sDFT/SCF iteration was one minute. For the basis-set sDFT calculation we used the same integration grid.

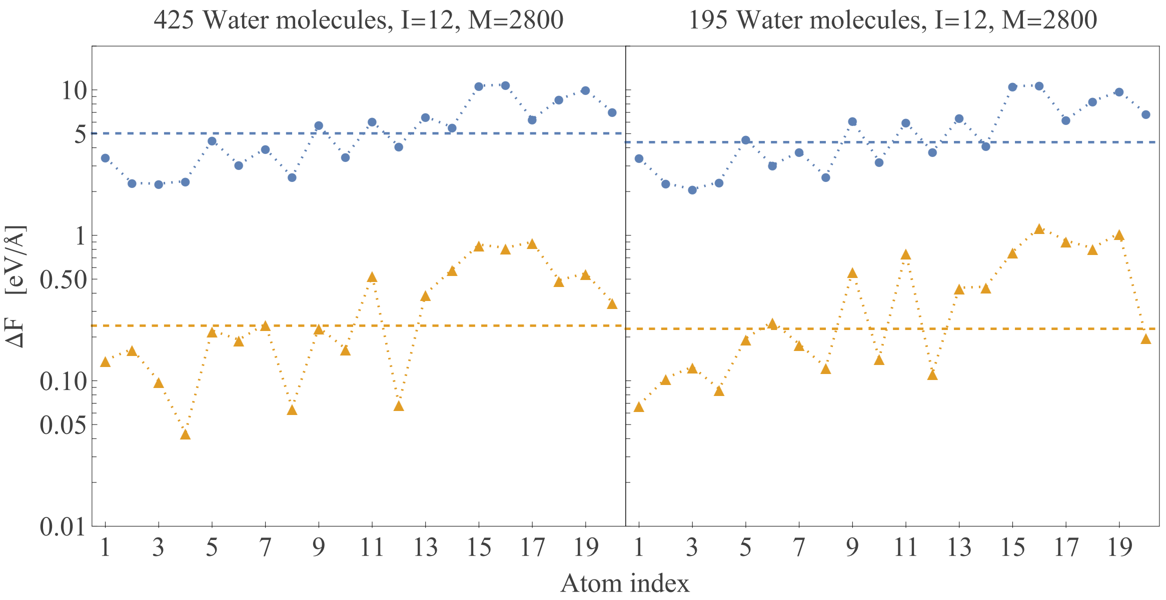

Figure S2. The statistical errors in the sDFT forces acting on the 20 nitrogen atoms in two solvated-TrpZip2 systems (using 425 solvating water molecules on the left and 195 on the right). Forces for both systems were calculated using $I = 12$ stochastic vectors. For each Nitrogen atom, we show the uncertainty σ_C (blue dots), and the estimate in the bias $\Delta\rho_C$ (orange triangles), see Eqs. $27 - 28$ in manuscript. The dotted lines connecting the markers are presented as a guide for the eye, while the dashed horizontal lines are medians over all atoms of σ_C and $\Delta\rho_C$.

	Relative speed of Hamiltonian operation	Relative Chebyshev expansion length	Relative speed	Relative statistical error in force	Relative required number of CPUs	Relative wall-time	
	v_H	N_C	$v = \frac{v_H}{N_C}$	δf	$N_p = \delta f^2$	Running on the same number of processors N_p/v	Running on N_p times more processors $1/v$
STO-3G	40	0.08	500	1.4	2	0.004	2×10^{-3}
6-31G	4	0.13	31	4.6	21	0.700	3×10^{-2}
Real space	1	1	1	1	1	1	1

Table S1. Data for comparing speeds and wall times for real-space and basis-set calculations

Here are the results of our analysis (summarized in Table S1): the Hamiltonian operation for STO-3G and 6-31G are a factor 40 and 4 respectively faster than that of the real-space grid. Furthermore, the energy range of the basis sets is much smaller, so the Chebyshev expansion length of STO-3G and 6-31G are a factor 13 and 8 shorter than that of the real space grid. As a result, the operation of the DM on a vector in STO-3G and 6-31G are a factor of about 500 and 30 faster respectively than in the real-space grid calculation. Therefore, the calculation of a single SCF iteration is much faster in the basis set calculations than in the real-space grid.

Next, we considered the fluctuation error in both calculations. Surprisingly, we found, that the standard deviation of a typical force component on Si, for the STO-3G and the 6-31G basis sets are larger by a factor 1.4 and 4.6 respectively than that for the real-space grid. This means that for a given standard deviation goal, the basis set calculations will require more CPUs by factors of about $N_p = 1.4^2 \approx 2$ and $N_p = 4.6^2 \approx 21$, respectively, than the grid calculation.

Summarizing, STO-3G calculations can be very fast relative to the real-space grid's, however, it is well known that STO-3G is not accurate enough to be a reliably useful basis. When run with the same number of processors, the overall numerical effort of the 6-31G basis is comparable to that of the real-space grid calculation. However, if one can allocate a factor of $N_p \approx 21$ more processors to the 6-31G calculation than needed by the real-space grid, the

overall speed of the former will be 30 times faster.

---

[1] L. Kleinman and D. M. Bylander, Phys. Rev. Lett. **48**, 1425 (1982).
[2] A. Papoulis and S. U. Pillai, *Probability, random variables, and stochastic processes*, 4th ed. (McGraw-Hill, Boston, 2002).
[3] E. Arnon, E. Rabani, D. Neuhauser, and R. Baer, The Journal of Chemical Physics **146**, 224111 (2017).
